# The efficiency of neoadjuvant chemotherapy in colon cancer with mismatch repair deficiency

**DOI:** 10.1002/cam4.5076

**Published:** 2022-07-29

**Authors:** Wu Yunlong, Liu Tongtong, Zeng Hua

**Affiliations:** ^1^ Department of General Surgery Beijing Chao‐Yang Hospital, Capital Medical University Beijing China; ^2^ Department of Radiology Beijing Chao‐Yang Hospital, Capital Medical University Beijing China; ^3^ Department of Pathology, National Cancer Center/National Clinical Research Center for Cancer/Cancer Hospital Chinese Academy of Medical Sciences and Peking Union Medical College Beijing China

**Keywords:** colon cancer, mismatch repair deficiency, neoadjuvant chemotherapy, response

## Abstract

Colon cancers with mismatch repair deficiency (dMMR) have specific clinicopathologic characteristics compared with mismatch repair proficiency (pMMR); however, the effect of MMR status on the efficiency of neoadjuvant chemotherapy (NCT) remains unclear. In our study, 439 dMMR and 26 pMMR colon cancer patients with or without NCT from 2010 to 2017 were retrospectively collected. Clinicopathological features, treatment response, and survival were compared between different groups. In the dMMR group, patients with NCT were likely to have higher CEA (abnormal CEA: 51.6% vs. 17.4%, *p* < 0.001), more multiorgan resection (38.7% vs. 16.8%, *p* = 0.006), and larger postoperative tumor diameter (7.26 vs. 6.21, *p* = 0.033) than those without NCT, but nearly half of cT4b patients who had NCT (42.9%, 9/21) avoid multiorgan resection. pT4 stage (HR, 14.97; 95% CI, 1.88–118.92; *p* = 0.010), number of positive lymph nodes (HR, 1.17; 95% CI, 1.09–1.26; *p* < 0.001), and tumor deposit (HR, 6.73; 95% CI, 2.08–21.74; *p* = 0.001) were independent prognosis factors of disease‐free survival (DFS). For the advanced tumor subset, there is no significant difference between patients with or without NCT for OS (*p* = 0.13) and DFS (*p* = 0.11), although the survival rate of NCT was higher than non‐NCT patients. Moreover, tumor regression grade was similar between dMMR and pMMR patients who had NCT. This study showed that NCT was more likely to be employed in dMMR patients with advanced tumors and may reduce the rate of multiorgan resection for cT4b dMMR patients. More large‐scaled researches are needed to further explore if MMR status could predict the efficacy of neoadjuvant chemotherapy in patients with colon cancer.

## Graphical abstract

NCT could had its efficiency in patients with dMMR colon cancer regarding to tumor regression grade and the rate of multiorgan resection. Large‐scaled prospective researches are needed to verify our results and further explore more individual systemetic treatment for patients with colon cancer.

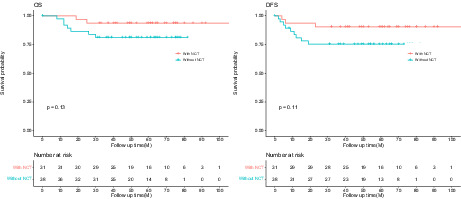

## 1 INTRODUCTION

According to the status of the mismatch repair (MMR) system, colorectal cancer (CRC) patients can be divided into two different molecular subtypes, including proficient mismatch repair (pMMR), and deficient mismatch repair (dMMR).^1^ MMR system helps to maintain genome stability, the deficiency of the function can lead to (also means MMR protein deficient) genetic errors accumulation, causing microsatellite instability (MSI) and tumorigenesis, which account for about 15% of CRC.^2^, ^3^, ^4^ Clinically, dMMR/MSI CRC is always characterized by proximal location, poor differentiation, early TNM stage, and good survival.^2^, ^5^, ^6^, ^7^ In addition, studies have revealed that MSI tumors have much more infiltrating lymphocytes than microsatellite stability (MSS),^8^, ^9^ which is considered as one of the primary causes for a good response to immunotherapy.^10^, ^11^

For colon cancer, surgery and chemotherapy are the main treatments. High‐risk stage II (only for pMMR/MSS) and stage III (both pMMR/MSS and dMMR/MSI) colon cancer patients are commonly recommended to receive adjuvant chemotherapy after radical surgery to reduce the risk of recurrence.^12^, ^13^ However, several landmark researches have found that stage II dMMR/MSI colon cancer does not benefit from fluorouracil‐based adjuvant chemotherapy,^13^, ^14^ indicating different drug susceptibility between dMMR/MSI and pMMR/MSS patients. Neoadjuvant chemotherapy (NCT) has been proved feasibility in locally advanced colon cancer (cT3‐4N0‐2M0) with acceptable toxicity and marked downstaging and significantly reduced rate of incomplete surgery resection which is conducted by the FOxTROT Collaborative Group.^15^ Thereafter, the NCCN guideline recommends colon cancer patients with bulky nodal disease or cT4 to receive NCT.^16^ Further results of the research have been reported recently that for patients with NCT, there was a trend toward less recurrent/residual disease within 2 years (HR 0.77, 95% CI 0.56–1.06), but did not reach significance (*p* = 0.11).^17^ One strategy to improve the efficacy of NCT is predicting drug sensitivity to improve the accuracy of treatment, in other words, patients with different phenotypes and characteristics may have different treatment efficacy and should receive different comprehensive regimens to achieve the best outcomes.

At the 2020 ASCO Annual Meeting, the FOxTROT Collaborative Group exhibited in the form of a meeting abstract that NCT brings significantly less moderate or greater histological tumor regression (7% (8/115) versus 23% (128/553), *p* < 0.001) in dMMR patients than pMMR, and NCT (FOLFOX with or without panitumumab) could only improve survival in pMMR patients.^18^ With the gradually increased application of NCT in locally advanced colon cancer patients, it is necessary to further explore if MMR status had an impact on the effect of NCT in colon cancer, given the biological and clinicopathologic difference between dMMR and pMMR patients. This study aims to explore the response to NCT and survival in dMMR colon cancer, speculating that NCT would also bring benefits to locally advanced dMMR colon cancer patients.

## 2 METHOD

### 2.1 Study population

The data of colorectal cancer patients who were diagnosed pathologically and underwent radical surgery at the National Cancer Center were collected from two independent retrospective study cohorts, including the dMMR cohort (patient with or without NCT) collected from 2011 to 2017 and the pMMR cohort (all patients received NCT) collected from 2010 to 2014. We excluded the patients with multiple primary tumors, distant metastasis, neoadjuvant chemoradiotherapy/radiotherapy, incomplete clinical information, and those who were rectal cancer. Furthermore, patients with pathologic complete response were not included in the cohort because MMR status was undetectable with no tumor residue. The data of colon cancer patients who met the standard was collected. And finally, we built two databases: one is composed of dMMR patients with or without NCT, and the other one is composed of pMMR patients who were clinically staged as cT4N+ and received NCT.

### 2.2 Pathological examination and MMR status determination

After the operation, all tumor tissues were formalin‐fixed and paraffin‐embedded. The specimens were reviewed by professional gastrointestinal pathologists. RLNs were carefully searched in each resected specimen. If the number of examined RLNs was <12, another attempt was made to identify additional lymph nodes. The response to NCT was evaluated using the Dworak tumor regression grade (TRG),^19^ which is the most commonly used grade system in our cancer center. TRG was scored as follows: Grade 0:no regression; Grade 1: dominant tumor mass with obvious fibrosis; Grade 2: dominantly fibrotic changes with few tumor cells; Grade 3: very few (difficult to find microscopically) tumor cells in the fibrotic tissue; Grade 4: no viable tumor cells (total regression or response).

Immunohistochemistry (IHC) staining for MMR proteins was performed on all specimens, and an automated IHC/ISH slide staining instrument (The DAKO AutostainerLink48) was used to stain the formalin‐fixed, paraffin‐embedded, 5‐μm sections according to the manufacturer's instructions. Clonal antibodies against MLH1 (ES05; DAKO, Denmark), MSH2 (FE11; DAKO, Denmark), MSH6 (EP49; DAKO, Denmark), and PMS2 (EP51; DAKO, Denmark) were used. The four proteins were all located in the nucleus and dMMR was considered when nuclear staining of any one of the four proteins was completely absent in tumor epithelial cells.

### 2.3 Treatment and outcome

The data of NCT, surgery and adjuvant chemotherapy regimens were collected. Clinical TNM stages (cT, cN, and cTNM) were assessed by contrast‐enhanced computed tomography before treatment. Tumor downstage was defined as a postoperative pathologic TNM stage lower than the clinical TNM stage. All the patients were recommended routine follow‐up consisting of physical examination, biological tests (including serum carcinoembryonic antigen), and computed tomography scan (or ultrasonography) every 3 months for the first 2 years and every 6 months for the following 3 years. The patient who did not go back to our hospital and the lost track was considered lost to follow‐up. The data were updated in October 2021.

### 2.4 Statistical analysis

Baseline clinicopathological variables were described as means and standard deviation for continuous variables and percentages for qualitative variables. Differences in characteristics between subgroups were assessed using the two independent sample *t*‐test, chi‐square test, or Fisher's exact test. Disease‐free survival (DFS) was defined as the time elapsed from surgery to the first recurrence. Overall survival (OS) was defined as the time elapsed from surgery to death. The occurrence of non‐colorectal tumors was disregarded. Survival curves were estimated using the Kaplan–Meier method and described using a 95% confidence interval (CI). Univariable and multivariable Cox proportional regression hazard models were performed to estimate hazard ratios (HRs) and 95% CIs. All variables with *p* < 0.05 in the univariable model were included in the multivariable model, and the R package *stepAIC* was used to further optimize the model by AIC. Hazard proportionality was tested by using the function *cox.zph* and if *p* > 0.05, then we thought that the model fit the hypothesis. All *p*‐values were two‐tailed and considered statistically significant if *p* < 0.05. All statistical analyses were performed using R software, version 4.1.2.

## 3 RESULTS

### 3.1 Characteristics of the dMMR Cohort

**FIGURE 1:**
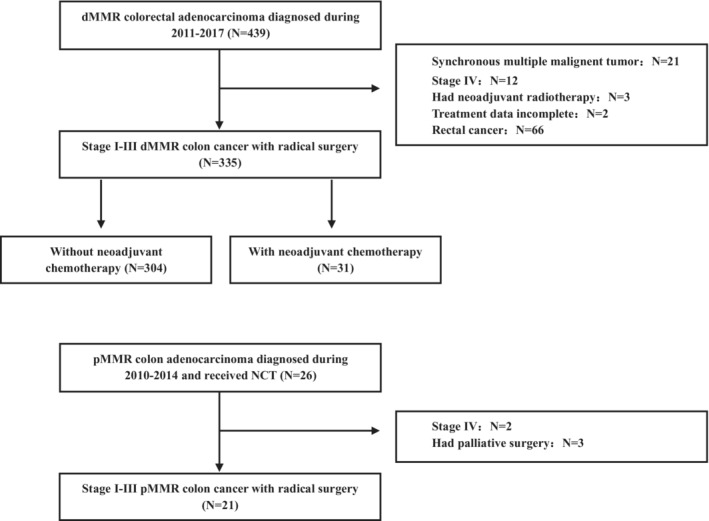
Cohort selection criteria for dMMR and pMMR colorectal cancer patients from 2010 to 2017.

Totally, 439 dMMR colorectal cancer were selected and reviewed, and 335 stage I–III patients with radical surgery were finally included in this study: NCT group (*n* = 31) and non‐NCT group (*n* = 304) (Figure 1). In the NCT group, the reasons for NCT include reduced tumor burden to reduce the scope of surgical resection and increased complete resection rate (cT4b: 67.7%, 21/31), multiple local lymph node metastases (29.0%, 9/31), and unknown (3.2%, 1/31). The NCT regimen including CAPEOX (38.7%, 12/31), FOLFOX (25.8%, 8/31), FOLFIRI (6.5%, 2/31), FOLFOXIRI (16.1%, 5/31), and others (12.9%, 4/31). Four of them (12.9%, 4/31) also received targeted drugs. As shown in Table 1, the median age was 55 (19–83 years), with 193 (57.6%) males and 142 (42.4%) females. dMMR patients who had NCT were likely to have higher CEA (abnormal CEA: 51.6% vs. 17.4%, *p* < 0.001), more multiorgan resection (38.7% vs. 16.8%, *p* = 0.006), and larger tumor diameter (7.26 vs. 6.21, *p* = 0.033). On the contrary, there was no significant association between NCT and other clinicopathologic characteristics, such as differentiation, lymphovascular invasion, nerve invasion, tumor deposit, total number of lymph nodes (TLN), negative lymph nodes (NLN), and positive lymph nodes (PLN). Although in the NCT group, all the patients' clinical T stage was cT4, 90.3% of the patients' clinical N stage were cN+, the postoperative pathological stage is not significantly different between NCT and non‐NCT groups and there were still more patients in the NCT group received adjuvant chemotherapy (mainly consisted of CAPEOX or FOLFOX which is not shown) compared with the non‐NCT group (87.1% vs. 50.3%, *p* < 0.001).

**TABLE 1:** Baseline characteristics of dMMR patients with (*N* = 31) or without NCT (*N* = 335).

	Total (*N* = 335)	Yes (*N* = 31)	No (*N* = 304)	*p* value
Age				0.671
Mean (*SD*)	54.7 (13.2)	55.7 (10.9)	54.7 (13.4)	
Median [Min, Max]	55.0 [19.0, 83.0]	57.0 [35.0, 75.0]	54.0 [19.0, 83.0]	
Sex				0.314
Male	193 (57.6%)	21 (67.7%)	172 (56.6%)	
Female	142 (42.4%)	10 (32.3%)	132 (43.4%)	
CEA				<0.001
Abnormal	69 (20.6%)	16 (51.6%)	53 (17.4%)	
Normal	197 (58.8%)	12 (38.7%)	185 (60.9%)	
Missing	69 (20.6%)	3 (9.7%)	66 (21.7%)	
CA19.9				0.197
Abnormal	40 (11.9%)	7 (22.6%)	33 (10.9%)	
Normal	227 (67.8%)	21 (67.7%)	206 (67.8%)	
Missing	68 (20.3%)	3 (9.7%)	65 (21.4%)	
Laparoscope				0.090
Laparotomy	111 (33.1%)	15 (48.4%)	96 (31.6%)	
Laparoscope	224 (66.9%)	16 (51.6%)	208 (68.4%)	
Multiorgan resection				0.006
Yes	63 (18.8%)	12 (38.7%)	51 (16.8%)	
No	272 (81.2%)	19 (61.3%)	253 (83.2%)	
Diameter				0.033
Mean (*SD*)	6.30 (2.64)	7.26 (3.39)	6.21 (2.54)	
Median [Min, Max]	6.00 [1.50, 18.0]	7.00 [2.50, 18.0]	6.00 [1.50, 15.0]	
Subtype				0.096
Swell	172 (51.3%)	11 (35.5%)	161 (53.0%)	
Ulceration+Infiltration	163 (48.7%)	20 (64.5%)	143 (47.0%)	
Histology				0.085^a^
Aden	296 (88.4%)	25 (80.6%)	271 (89.1%)	
Muci	26 (7.8%)	6 (19.4%)	20 (6.6%)	
Sign	1 (0.3%)	0 (0%)	1 (0.3%)	
Mixed	12 (3.6%)	0 (0%)	12 (3.9%)	
Differentiation				0.133^a^
Well	14 (4.2%)	3 (9.7%)	11 (3.6%)	
Moderate	185 (55.2%)	14 (45.2%)	171 (56.3%)	
Poor	128 (38.2%)	14 (45.2%)	114 (37.5%)	
Missing	8 (2.4%)	0 (0%)	8 (2.6%)	
Lymphovascular invasion				0.908
Yes	62 (18.5%)	5 (16.1%)	57 (18.8%)	
No	273 (81.5%)	26 (83.9%)	247 (81.3%)	
Nerve invasion				1.000^a^
Yes	38 (11.3%)	3 (9.7%)	35 (11.5%)	
No	297 (88.7%)	28 (90.3%)	269 (88.5%)	
Tumor deposit				1.000^a^
Yes	12 (3.6%)	1 (3.2%)	11 (3.6%)	
No	323 (96.4%)	30 (96.8%)	293 (96.4%)	
Local invasion				0.152^a^
Yes	15 (4.5%)	3 (9.7%)	12 (3.9%)	
No	320 (95.5%)	28 (90.3%)	292 (96.1%)	
TLN				0.091
Mean (*SD*)	34.6 (16.5)	39.4 (23.1)	34.1 (15.7)	
Median [Min, Max]	31.0 [7.00, 104]	32.0 [13.0, 97.0]	31.0 [7.00, 104]	
NLN				0.065
Mean (*SD*)	33.8 (16.8)	39.1 (23.0)	33.2 (16.0)	
Median [Min, Max]	30.0 [6.00, 104]	31.0 [13.0, 97.0]	30.0 [6.00, 104]	
PLN				0.256
Mean (*SD*)	0.863 (2.61)	0.355 (0.839)	0.914 (2.72)	
Median [Min, Max]	0 [0, 24.0]	0 [0, 4.00]	0 [0, 24.0]	
pT				0.409^a^
T1–T3	216 (64.5%)	17 (54.8%)	199 (65.5%)	
T4	119 (35.5%)	14 (45.2%)	105 (34.5%)	
pN				0.648^a^
N0	246 (73.4%)	23 (74.2%)	223 (73.4%)	
N1	64 (19.1%)	7 (22.6%)	57 (18.8%)	
N2	25 (7.5%)	1 (3.2%)	24 (7.9%)	
PTNM				0.286^a^
I	24 (7.2%)	0 (0%)	24 (7.9%)	
II	222 (66.3%)	23 (74.2%)	199 (65.5%)	
III	89 (26.6%)	8 (25.8%)	81 (26.6%)	
Adjuvant chemotherapy				<0.001
Yes	180 (53.7%)	27 (87.1%)	153 (50.3%)	
No	150 (44.8%)	4 (12.9%)	146 (48.0%)	
Missing	5 (1.5%)	0 (0%)	5 (1.6%)	

Abbreviations: Aden, adenocarcinoma; Muci, mucinous adenocarcinoma; Sign, signet‐ring cell carcinoma; Mixed, could not be classified.

^a^ Fisher's exact test.

### 3.2 Prognosis of the dMMR Cohort

**FIGURE 2:**
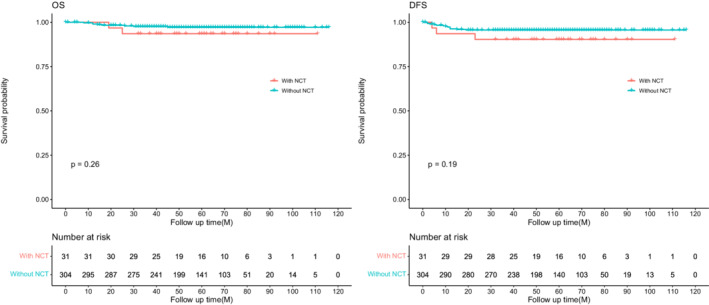
Progression‐free survival (PFS) and overall survival (OS) in dMMR colon cancer patients with or without neoadjuvant therapy (NCT).

**FIGURE 3:**
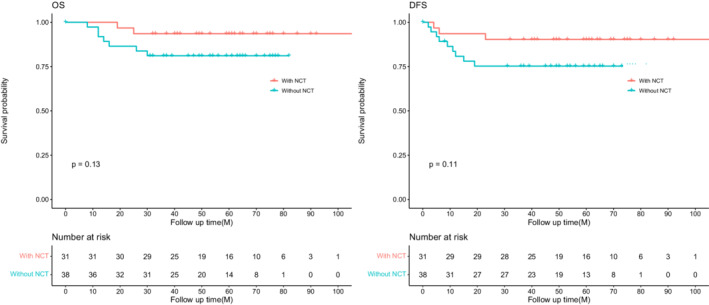
Progression‐free survival (PFS) and overall survival (OS) in locally advanced dMMR colon cancer patients with or without neoadjuvant therapy (NCT).

The median follow‐up time was 56 months (IQR, 42–74 months) and 8.4% (28/335) were lost to follow‐up. The 3‐year OS and DFS were 93.5%, 90.3%, and 97.6%, 95.6% for NCT and non‐NCT groups, respectively. And the log‐rank test revealed no significance between the two groups for OS (*p* = 0.26) and DFS (*p* = 0.19) (Figure 2). In univariate Cox analysis, CA19‐9, multiorgan resection, lymphovascular invasion, nerve invasion, tumor deposit, local invasion, pathologic T4, PLN, and adjuvant chemotherapy were associated with DFS (*p* < 0.05), but NCT did not reach significance (*p* = 0.204). These candidate variables (still including NCT) were involved in the multivariate Cox regression model, NCT did not improve DFS for dMMR patients (HR, 3.15; 95% CI, 0.81–12.15; *p* = 0.096), but pathologic T4 stage (HR, 14.97; 95% CI, 1.88–118.92; *p* = 0.010), PLN (HR, 1.17; 95% CI, 1.09–1.26; *p* < 0.001) and tumor deposit (HR, 6.73; 95% CI, 2.08–21.74; *p* = 0.001) were still significantly associated with DFS. Differentiation was not involved in the model because all the patients with well‐differentiated tumor had no local recurrence or metastasis (Table 2). Furthermore, clinical locally advanced patients (with NCT: cT4N+; without NCT: pT4N+) were selected to analyze the effect of NCT on survival, and there is no significant difference between patients with or without NCT for OS (*p* = 0.13) and DFS (*p* = 0.11), although the survival rate of NCT was higher than non‐NCT patients (Figure 3).

**TABLE 2:** Univariate and multivariate Cox regression analysis for DFS in dMMR patients.

	Univariate			Multivariate		
	HR	95% CI	*p* value	HR	95% CI	*p* value
Age	1.01	0.97–1.05	0.589			
Sex						
Male versus Female	1.60	0.56–4.61	0.383			
CEA						
Abnormal versus Normal^a^	2.47	0.83–7.35	0.104			
CA19‐9						
Abnormal versus Normal^b^ ^,^ ^d^	5.09	1.71–15.13	0.003			
Laparoscope						
Laparoscope versus Laparotomy	0.39	0.14–1.03	0.058			
Tumor size	1.00	0.84–1.21	0.963			
NACT						
Yes versus No	2.26	0.64–7.92	0.204	3.15	0.81–12.15	0.096
Multi‐organ resection						
Yes versus No	4.50	1.69–11.99	0.003			
Subtype						
Ulceration+Infiltration versus Swell	2.32	0.81–6.68	0.119			
Histology						
Adenocarcinoma versus other^c^	0.57	0.16–2.00	0.378			
Lymphovascular invasion						
Yes versus No	3.69	1.37–9.90	0.010			
Nerve invasion						
Yes versus No	3.94	1.37–11.34	0.011	2.42	0.81–7.28	0.116
Tumor deposit						
Yes versus No	10.27	3.31–31.88	<0.001	6.73	2.08–21.74	0.001
Local invasion						
Yes versus No	8.36	2.69–25.95	<0.001			
pT						
pT4 versus pT1‐3	13.27	3.02–58.42	<0.001	14.97	1.88–118.92	0.010
TLN	0.99	0.96–1.02	0.493			
NLN	0.97	0.93–1.01	0.967			
PLN	1.18	1.11–1.26	<0.001	1.17	1.09–1.26	<0.001
Adjuvant chemotherapy						
Yes versus No	11.94	1.57–90.82	0.017			

^a^ Normal: CEA ≤ 5 ng/ml; abnormal: CEA > 5 ng/ml.

^b^ Normal: CA19‐9 ≤ 37 U/ml; abnormal: CA19‐9 > 37 U/ml.

^c^ “Other” includes mucinous adenocarcinoma, signet‐ring cell carcinoma and mixed type which could not be classified.

^d^ CA19‐9 did not involve in multivariate COX regression analysis because a large proportion of patients did not test CA19‐9 before NCT or surgery.

### 3.3 The baseline information compared between dMMR and pMMR colon cancer patients with NCT

Data of 21 cT4N+ pMMR colon cancer patients with NCT was collected. The reasons for NCT includes: reduced tumor burden to increase complete resection rate (cT4b: 38.1%, 8/21) and multiple local lymph node metastasis (61.9%, 13/21). The NCT regimen including CAPEOX (42.9%, 9/21), FOLFOX (42.9%, 9/21), FOLFIRI (9.5%, 2/21), and others (4.8%, 1/21). Three of them (14.3%, 3/21) also received targeted drugs. As shown in Table 3, there was no significant difference between age, sex, CEA, CA9‐9, cT, and cN, which indicates a similar demographic and tumoral clinical stage between dMMR and pMMR. On the aspect of postoperative pathology, most risk factors were not relevant to MMR status, except differentiation (*p* = 0.024) and pTNM stage (*p* = 0.029). Furthermore, dMMR patients with NCT still had higher TLN (39.4 vs. 26.6, *p* = 0.026), NLN (39.1 vs. 25.3, *p* = 0.017), and less PLN (0.36 vs. 1.33, *p* = 0.015) than pMMR patients.

**TABLE 3:** Baseline characteristics of dMMR (*N* = 31) and pMMR (*N* = 21) colon cancer patients with NCT.

	Total (*N* = 52)	dMMR (*N* = 31)	pMMR (*N* = 21)	*p* value
Age				0.627
Mean (*SD*)	55.1 (11.3)	55.7 (10.9)	54.1 (12.0)	
Median [Min, Max]	57.0 [29.0, 75.0]	57.0 [35.0, 75.0]	57.0 [29.0, 74.0]	
Sex				0.627
Male	33 (63.5%)	21 (67.7%)	12 (57.1%)	
Female	19 (36.5%)	10 (32.3%)	9 (42.9%)	
CEA				0.718
Abnormal	25 (48.1%)	16 (51.6%)	9 (42.9%)	
Normal	22 (42.3%)	12 (38.7%)	10 (47.6%)	
CA19.9				0.194
Abnormal	8 (15.4%)	7 (22.6%)	1 (4.8%)	
Normal	38 (73.1%)	21 (67.7%)	17 (81.0%)	
cT				*
cT3	0 (0%)	0 (0%)	0 (0%)	
cT4	52 (100%)	31 (100%)	21 (100%)	
cN				0.264^a^
cN0	3 (5.8%)	3 (9.7%)	0 (0%)	
cN+	49 (94.2%)	28 (90.3%)	21 (100%)	
cTNM				0.264^a^
II	3 (5.8%)	3 (9.7%)	0 (0%)	
III	49 (94.2%)	28 (90.3%)	21 (100%)	
Laparoscope				0.309
Laparotomy	29 (55.8%)	15 (48.4%)	14 (66.7%)	
Laparoscope	23 (44.2%)	16 (51.6%)	7 (33.3%)	
Diameter				0.001
Mean (*SD*)	6.08 (3.17)	7.26 (3.39)	4.32 (1.73)	
Median [Min, Max]	5.65 [1.50, 18.0]	7.00 [2.50, 18.0]	4.00 [1.50, 8.50]	
Subtype				1.000
Swell	18 (34.6%)	11 (35.5%)	7 (33.3%)	
Ulceration + Infiltration	34 (65.4%)	20 (64.5%)	14 (66.7%)	
Histology				0.070^a^
Aden	46 (88.5%)	25 (80.6%)	21 (100%)	
Muci	6 (11.5%)	6 (19.4%)	0 (0%)	
Sign	0 (0%)	0 (0%)	0 (0%)	
Mixed	0 (0%)	0 (0%)	0 (0%)	
Differentiation				0.024^a^
Well	6 (11.5%)	3 (9.7%)	3 (14.3%)	
Moderate	29 (55.8%)	14 (45.2%)	15 (71.4%)	
Poor	16 (30.8%)	14 (45.2%)	2 (9.5%)	
Lymphovascular invasion				0.414
Yes	6 (11.5%)	5 (16.1%)	1 (4.8%)	
No	46 (88.5%)	26 (83.9%)	20 (95.2%)	
Nerve invasion				0.388
Yes	3 (5.8%)	3 (9.7%)	0 (0%)	
No	49 (94.2%)	28 (90.3%)	21 (100%)	
Tumor deposit				1.000
Yes	2 (3.8%)	1 (3.2%)	1 (4.8%)	
No	50 (96.2%)	30 (96.8%)	20 (95.2%)	
Local invasion				0.577
Yes	7 (13.5%)	3 (9.7%)	4 (19.0%)	
No	45 (86.5%)	28 (90.3%)	17 (81.0%)	
TLN				0.026
Mean (*SD*)	34.3 (20.5)	39.4 (23.1)	26.6 (13.1)	
Median [Min, Max]	26.0 [9.00, 97.0]	32.0 [13.0, 97.0]	24.0 [9.00, 66.0]	
NLN				0.017
Mean (*SD*)	33.5 (20.7)	39.1 (23.0)	25.3 (13.2)	
Median [Min, Max]	25.5 [8.00, 97.0]	31.0 [13.0, 97.0]	21.0 [8.00, 66.0]	
PLN				0.015
Mean (*SD*)	0.750 (1.44)	0.360 (0.839)	1.33 (1.91)	
Median [Min, Max]	0 [0, 7.00]	0 [0, 4.00]	0 [0, 7.00]	
pT				0.212^a^
T1	0 (0%)	0 (0%)	0 (0%)	
T2	2 (3.8%)	0 (0%)	2 (9.5%)	
T3	29 (55.8%)	17 (54.8%)	12 (57.1%)	
T4	21 (40.4%)	14 (45.2%)	7 (33.3%)	
pN				0.106^a^
N0	33 (63.5%)	23 (74.2%)	10 (47.6%)	
N1	15 (28.8%)	7 (22.6%)	8 (38.1%)	
N2	4 (7.7%)	1 (3.2%)	3 (14.3%)	
pTNM				0.029^a^
I	1 (1.9%)	0 (0%)	1 (4.8%)	
II	32 (61.5%)	23 (74.2%)	9 (42.9%)	
III	19 (36.5%)	8 (25.8%)	11 (52.4%)	
Adjuvant chemotherapy				0.138
Yes	48 (92.3%)	27 (87.1%)	21 (100%)	
No	4 (7.7%)	4 (12.9%)	0 (0%)	

Abbreviations: Aden, adenocarcinoma; Mixed, could not be classified; Muci, mucinous adenocarcinoma; Sign, signet‐ring cell carcinoma.

^a^ Fisher's exact test.

* Did not do analysis.

### 3.4 The efficacy of NCT in dMMR and pMMR colon cancer patients

As shown in Table 4, 38.7% dMMR and 23.8% pMMR patients underwent multiorgan resection after NCT. Most tumor regression grades between the two groups were mild (dMMR vs. pMMR: 64.5% vs. 47.6%) and moderate (dMMR vs. pMMR: 16.1% vs. 28.6%). Similarly, more than half of the dMMR patients experienced a downstage, and is comparable to that of pMMR (64.5% vs. 47.6%). In addition, the 3‐year DFS and OS were 95.2% and 97.0% in dMMR patients, while they were 76.2% and 85.7% in pMMR patients.

**TABLE 4:** The effect of NCT between dMMR (*N* = 31) and pMMR (*N* = 21) colon cancer patients

	Total (*N* = 52)	dMMR (*N* = 31)	pMMR (*N* = 21)
Multi‐organ resection			
Yes	17 (32.7%)	12 (38.7%)	5 (23.8%)
No	35 (67.3%)	19 (61.3%)	16 (76.2%)
Downstage			
Yes	30 (57.7%)	20 (64.5%)	10 (47.6%)
No	22 (42.3%)	11 (35.5%)	11 (52.4%)
TRG			
Grade 0	5 (9.6%)	2 (6.5%)	3 (14.3%)
Grade 1	30 (57.7%)	20 (64.5%)	10 (47.6%)
Grade 2	11 (21.2%)	5 (16.1%)	6 (28.6%)
Grade 3	2 (3.8%)	0 (0%)	2 (9.5%)

## 4 DISCUSSION

The feasibility of NCT has been demonstrated by the FOxTROT Collaborative Group in locally advanced colon cancer.^15^, ^17^ Thereafter, the NCCN guideline recommends colon cancer patients with a bulky nodal disease or cT4 to receive NCT.^16^ In 2020, the FOxTROT represented in the form of a meeting abstract that the effect of NCT could be different based on MMR status.^18^ And our study further comprehensively analyses the effect of NCT in dMMR colorectal cancer patients. We found that in the dMMR group, patients with NCT were likely to have higher CEA and larger postoperative tumor size and a resultant higher rate of multi‐organ resection than those without NCT, but there is no significant difference regarding pTNM stage, TLN, PLN, and other common pathologic risk factors. For survival, pathologic T4 stage, PLN, and tumor deposit are independent prognostic factors but not NCT, although in the locally advanced dMMR subgroup (cT4N+ patients with NCT and pT4N+ patients without NCT), patients who received NCT showed a trend for better survival. Compared with pMMR patients with NCT, dMMR patients who received NCT still showed more poor differentiation, higher NLN, less PLN, and comparable tumor regression grade.

To achieve tumor response and shrinkage is the primary objection to neoadjuvant therapy, aiming to increase radical resectability.^15^, ^20^ Furthermore, patients with favorable response to neoadjuvant therapy are likely to have a better long‐term outcome than those with insignificant response.^21^, ^22^ In colon cancer, the FOxTROT group revealed a trend toward less recurrence within 2 years for patients who received NCT (*p* = 0.11)^17^ with 2.1% (2/94) complete response and 28.7% (27/94) moderate to the marked response. In this study, 80.6% (25/31) and 64.5% (20/31) of dMMR patients with NCT had treatment response and downstage, respectively, which were similar to that of the pMMR group. Twenty‐one dMMR patients were evaluated as cT4b before treatment and nearly half of them (42.9%, 9/21) avoid multi‐organ resection after NCT. For survival analysis, we found that pT4 stage, positive lymph nodes, and tumor deposit but not NCT were independent prognosis factors of DFS (multivariate COX analysis for OS did not perform because the number of mortality events was too small). The relationship between MMR status and adjuvant chemotherapy has been explored,^12^, ^13^, ^14^, ^23^ and it is now generally accepted that 5‐FU‐based adjuvant chemotherapy does not improve survival in stage II dMMR patients, but stage III dMMR patients still benefited from adjuvant chemotherapy, especially for combined treatment. And the results of our study indicate that dMMR patients could also benefit from NCT treatment. In addition, it has been well explored that dMMR colon cancer patients always had better survival than pMMR,^2^, ^7^, ^24^ and in our study, the survival rate of dMMR patients with NCT is still higher than pMMR, but survival analysis was not performed because the limitation of sample size.

Besides primary tumor response, neoadjuvant treatment may also influence regional lymph nodes. In rectal cancer, neoadjuvant radiotherapy with or without chemotherapy resulted in a mean reduction of 3.9 TLN and 0.7 PLN which reflect the effect on downstaging.^25^ It is commonly believed that radiation could induce shrinkage and fibrosis, as well as lymphocyte depletion and replacement by adipocytes in regional lymph nodes.^26^ In colon cancer, NCT is also a major factor for inadequate lymph node retrieval.^27^ However, in the dMMR cohort, not only did NCT not reduce the number of lymph node harvest but also harvested higher TLN with less PLN in colon cancer patients, although not statistically significant (TLN:39.4 vs. 34.1, *p* = 0.091; PLN:0.355 vs. 0.914, *p* = 0.256). As we know, the number of lymph node harvest is related to the survival in colon cancer patients^28^, ^29^ which is proposed as a reflection of immune status. According to our result, we make a bold assumption that NCT leads to tumor death and may expose more tumor‐associated‐antigen which further activates antitumor immunity in dMMR patients. The exact mechanism between NCT and regional immunity in dMMR and pMMR colon cancer patients still needs further exploration.

Immunotherapy targeting programmed cell death protein 1 (PD‐1) or its ligand (PD‐L1) has achieved promising results in clinical trials of dMMR/MSI colorectal cancer patients. The phase II KEYNOTE‐016 study first revealed the relationship between MMR status and pembrolizumab (anti‐PD‐1) efficacy. For 28 treatment‐refractory progressive metastatic colorectal cancers, 0% (0/18) of pMMR and 40% (4/10) of dMMR patients experienced an immune‐related objective response.^10^ In 2020, a phase III trial further estimate the superior effect of pembrolizumab to 5‐FU‐based chemotherapy (with/without targeted drug) in stage IV dMMR/MSI colorectal cancer. The overall response rate was observed in 43.8% and 33.1% patients, respectively, and patients who had a response to pembrolizumab were more likely to have ongoing responses (83% vs. 35% at 24 months) and better progression‐free survival (16.5 vs. 8.2 months, *p* = 0.002).^11^ Nevertheless, the response rate of immunotherapy remains around 40% even in dMMR patients, thus enhancing treatment efficacy has become a research hotspot. Chemotherapy can promote tumor immunity by immunogenic cell death and disrupting the tumor immune escape.^30^ The combination of chemotherapy and immunotherapy may achieve a better therapeutic effect than monotherapy. In 2020, Dung T Le et al. enrolled 124 dMMR/MSI‐H CRC patients who were previously treated (FOLFOXIRI therapy with/without monoclonal antibody) and with unresectable or metastatic tumor, after receiving pembrolizumab, the objective response rate is more than 30% and the median overall survival is longer than 31.4 months with acceptable adverse events.^31^ Recently, an exploratory NICHE study involved early‐stage (stage I‐III) colon cancer patients with neoadjuvant immunotherapy and surgery found that all the dMMR patients (20/20) had a pathological response, including 19 major and 12 complete response, which indicate that neoadjuvant immunotherapy has the potential benefit for defined patients group.^32^ In our cohort, patients did not receive immunotherapy, but we reveal that NCT may also have an effect on dMMR tumors and provides a potential idea to improve the efficacy of treatment combined with immunotherapy and chemotherapy in dMMR colon patients.

For locally advanced (cT3‐4/cN+) rectal cancer, comprehensive treatment including neoadjuvant chemoradiotherapy, radical surgery, and adjuvant chemotherapy is the standard regimen,^33^ but the role of adjuvant chemotherapy for these patients is still not explicit and may be due to the inaccurate baseline staging, tumor downstaging and poor compliance to adjuvant chemotherapy.^34^, ^35^, ^36^ In colon cancer, the relationship between MMR status and adjuvant chemotherapy has been explored,^12^, ^13^, ^14^, ^23^and it is now generally accepted that 5‐FU‐based adjuvant chemotherapy can not improve survival in stage II dMMR patients, but stage III dMMR patients could benefit from adjuvant chemotherapy, especially for combined treatment.^16^ Similar to rectal cancer, we found that the majority of colon cancer patients with NCT received adjuvant chemotherapy after surgery, but adjuvant chemotherapy was associated with worse DFS in univariate analysis (*p* = 0.017) and not included in the multivariate model because patients with adjuvant chemotherapy always had advanced tumor stage. Thus, the effect of adjuvant chemotherapy for colon cancer patients after NCT is still unknown, and we believe that it is necessary to further explore particular colon cancer group who may indeed benefit from adjuvant chemotherapy after NCT.

This study has limitations inherent to retrospective observational research. The selection bias or potential confounding factors were uncontrolled. For example, although all of them were 5‐FU based, the regimen and cycles of NCT are not exactly the same, and the diagnostic time of pMMR and dMMR is also different (pMMR:2011–2017; dMMR:2010–2014) and quite a bit patients lost to follow up. Furthermore, only a few colon cancer patients received NCT with few final events in dMMR patients makes it difficult to obtain reliable statistical results, thus, we just showed the descriptive data but not comparison data between dMMR and pMMR groups to avoid a misleading conclusion. NCT was recommended for locally advanced colon cancer just in recent years and the cases are rare. However, as far as we know, this is still a relatively large‐scale cohort including colon cancer patients with dMMR and NCT. In addition, we did not collect and report the adverse events because the safety of NCT has been well discussed in other studies.^15^, ^17^ And IHC was used, rather than gold‐standard polymerase chain reaction (PCR) to assess MMR status, however, the study has revealed that IHC could provide comparable sensitivity and specificity to PCR.^37^

## 5 CONCLUSION

Neoadjuvant chemotherapy could have its efficiency in patients with dMMR colon cancer regarding tumor regression grade and the rate of multiorgan resection. Large‐scaled prospective researches are needed to verify our results and further explore more individual systematic treatment for patients with colon cancer.

## AUTHOR CONTRIBUTIONS

Wu Yunlong: Conception and design, data analysis and interpretation, writing, editing, and supervision; Liu Tongtong: Conception and design, collection and assembly of the data, data analysis and interpretation; Zeng Hua: Conception and design, collection and assembly of the data, data interpretation.

## FUNDING INFORMATION

There was no funding source for this study.

## CONFLICT OF INTEREST

None.

## ETHICS STATEMENT

None (this was a retrospective study that did not require ethical approval or participant consent).

## Data Availability

The data are available from the corresponding author upon reasonable request.
